# Supplementary material for: Field evaluation of sunflower as a potential trap crop of Lygus pratensis in cotton fields
Source: PLoS One. 2020 Aug 17;15(8):e0237318. doi: 10.1371/journal.pone.0237318 (PMC7430709; doi:10.1371/journal.pone.0237318)
Supplement: Attachment — Submitted filename: Response to Reviewers.docx [file pone.0237318.s001.docx]

Dear editors,

Thank you very much for your suggestions and comments on our manuscript. Those comments are invaluable for improving our paper. We have accordingly revised the manuscript carefully and hope that this new version could be accepted for publication in *PLOS ONE*. Our point-by-point responses are given below:

**I. Responses to the comments and suggestions of Referee**

1. You should describe insect sampling methods very carefully. “In each plot, five points were randomly selected and 10 plants were checked per point. On each plant, the number of *L. pratensis* adults was visually determined and recorded”. So, 50 plants were sampled in each field, right? Maybe a sampling diagram is better to show your method.

Also, you only counted the adults, or both adult and nymphs. There are also many nymphs in field. You did not show the nymph data in Figure 1. Why you ignore nymphs? You should add more information here.

**Reply: In term of the sampling methods, there is a writing error, we have corrected our mistake. Line108-109: changed to “In each plot, using five-point sampling method and 20 consecutive plants were checked each point”. Line106-109. Also, a sampling diagram (figure 1) is generated to show our method clearly. Line445-446.**

**Beginning in early June, as these first generation of *Lygus pratensis* nymphs become adults on spring host plants, they disperse to other host plants and then produce second generation there. In other words, before they attack the sunflower and cotton, no L. pratensis’ infestation was occurred on these two crops. Therefore, we only investigate the relative attractiveness of sunflowers and cottons to adults from entering period, ignore the nymphs here. Line110-114.**

2. About the field investigation, you have both sunflower and cottons. Do you investigate the abundance of *Lygus pratensis* in both crops? In figure 1, you showed the number of adults. Is that cotton? ZM 49 is cotton. I am confusing. I guess you investigate cotton. It makes no sense if you compare the differences between sunflowers and cotton.

**Reply: In our work, we indeed investigated the abundance of *Pratensis* in both sunflower and cottons. To clarify our meaning, we have now including the following in Figure 2 legends on Line 469 “Note：ZM49 is cotton, XKZ6 and SH363 are sunflower varieties.”**

**The aim of this research is to examine the potential of usage of sunflower as a trap crop for *L. pratensis* in cotton fields. Here, cotton is regarded as control group. Therefore, comparing the abundance of *L. pratensis* between sunflowers and cotton is requisite and meaningful. On Lines 166-169.**

3. You designed three treatments of planting time. In figure 3, do you combine all different time? It is inappropriate that you combine different treatment because late-planted sunflower seems more effective to control *Lygus pratensis*. I am also confusing that you had nymph data here. You never showed nymph data before.

**Reply: In figure 4, we only used the early-planted XKZ6 rather than combing all different time. The aim of the initial field-plot trials is to determine the suitable inter-planted sunflower strips, and the results have indicated the early-planted XKZ6 more effective to attract *Lygus pratensis* adults, in this context, this treatment was undoubtedly used for subsequent analysis. Lines. 172-176.**

**The emphases of these two treatments are different, in figure 2, first generation of L. pratensis as reproductive adults dispersed to other plants, and then produced second generation there. Therefore, we ignore nymphs here. In figure 4, to assess the role of sunflower strips on population dynamics of L. pratensis in cotton fields, the abundance of L. pratensis including adults and nymphs were investigated. 172-176.**

4. The discussion is too broad. Conclusion section. You said that early-planted XKZ6 sunflower strips (coupled with targeted insecticide sprays) to suppress field populations of bug. But I saw the abundance in cotton planted XKZ6 sunflower strips is highest based on Figure 1. Is that right?

**Reply: The objective of our work is to assess the relative attractiveness of sunflower (Helianthus annuus) to Lygus pratensis adults in local cotton plots. As shown in Figure 1, the abundance in cotton early-planted XKZ6 sunflower plots is highest, thus we concluded that establishment of sunflower strips could contribute to suppress field populations of bug in cotton crops. Our conclusion is coincident with figure 2.**

**The conclusion section was outlined in Discussion (Line203-210): “Our study validates the usage of sunflower as a trap crop for L. pratensis in cotton fields in southern Xinjiang, and describes the particular role of early-planted XKZ6 sunflower strips (coupled with targeted insecticide sprays) to suppress field populations of this mirid bug.”**

**T**hank you for your time and suggestion.

Kindest regards.

Yours,

Renfu Zhang

Institute of Plant Protection

Xinjiang Academy of Agricultural Sciences

No.403, Nanchang Road

Urumqi, Xinjiang 830091, China
