# Supplementary material for: Field evaluation of sunflower as a potential trap crop of Lygus pratensis in cotton fields
Source: PLoS One. 2020 Aug 17;15(8):e0237318. doi: 10.1371/journal.pone.0237318 (PMC7430709; doi:10.1371/journal.pone.0237318)
Supplement: Attachment — Submitted filename: Response to Reviewers.docx [file pone.0237318.s002.docx]

Dear editors,

Thank you very much for your attention and careful consideration to our manuscript. We appreciate reviewers very much for their constructive comments and suggestions on our manuscript.

We have carefully considered your and referee’s suggestion and comments and revised our manuscript according to these precious comments. Those comments are invaluable for improving our paper. We have accordingly revised the manuscript carefully and hope that this new version could be accepted for publication in *PLOS ONE* The main corrections and the responds to the comments point to point are given below:

**I. Responses to the comments and suggestions of Referee**

1. The title is not good enough. The following two titles are suggested: Field evaluation of sunflower as a potential trap crop of *Lygus pratensis* in cotton fields or Intercropping sunflower reduces the infestation and damage of *Lygus pratensis* in cotton fields.

**Reply: Thanks for the reviewer's suggestion, and the title has been changed to "Field evaluation of sunflower as a potential trap crop of *Lygus pratensis* in cotton fields"(Line 5-6)**

2. The introduction is not strong enough in the present version, and more latest literatures (published in the international journals) of intercropping effect on herbivorous pests and the underline mechanisms should be introduced.

**Reply: Thanks for the suggestion, and it has been** [**supplemented**](https://fanyi.so.com/#supplemented) **in the revised manuscript.**

3. The diagram in the current version is not the really diagram, and I made no sense from it. Please referred to other published papers.

**Reply: Thanks for the suggestion, and referred to the other published papers, we have been re-made the diagram in the revised manuscript.**

4. LSD was abandoned in the present. Turkey’stest is suggested to analyze your data.

**Reply: Thanks for the suggestion. Because the population dynamics of *Lygus pratensis* on cotton and sunflower are all showed seasonal changes, so the data analysis are as follows: Differences in the abundance of *L. pratensis* (including adults, nymphs, and total population, as three counts) in cotton and sunflower plots (2015-2016) or in cotton fields with/without sunflower strips (2016-2017) were analyzed by a repeated-measures analysis of variance (ANOVA) using SAS PROC MIXED, and the means were compared by the least significant difference test (LSD). The data were log10(n+1)-transformed before analysis. The mean abundances of *L. pratensis* adults in plots of cotton and sunflower during 2015-2016 were compared using one-way ANOVA followed by Tukey’s HSD for multiple comparisons. Mean abundances of *L. pratensis* population, the damage rate of leaves and bolls (arcsin-transformed), and the number of mature bolls in cotton fields with/without sunflower strips during 2016-2017 were compared through non-paired t-tests.**

5. The language of the paper should be polished.

**Reply: Thanks for the reviewer's suggestion, the language has been improved and polished in the revised manuscript.**

**II. Responses to the comments and suggestions of Referee 2**

1. Why did you use five-point sampling method? As Yao et al (2016) reported that the spatial distribution pattern *L. pratensis* were aggregated distribution.

**Reply: As Jiao et al (2012) reported that the spatial distribution pattern of *Apolygus lucorum* (Hemiptera: Miridae) is aggregated distribution. five-point sampling method is usually used for the investigation and sampling of *Apolygus lucorum* in fields (Wu et al, 2002; Lu et al, 2008; Lu et al, 2009), so we borrowed their method, using five-point sampling method in this paper.**

2. Please give location of the sample site when using five-point sampling？Especially when collecting data from trap-cropping field with two 1-m wide strips of sunflower on the opposite borders of the field, parallel to the cotton rows.

**Reply:** **In order to show our sampling method more intuitively, we made a sampling** diagram **(Figure 1b)**

3. As results of figure 2, the size of plant is important factor for *L. pratensis* to select plants. It is not very available to say the sunflower more attractive to cotton. Could you please show some other data such as comparing the attractive ability between same size cotton or sunflower.

**Reply: As you said, the size of plant is important factor for *L. pratensis* to select plants. In this paper, the two most common sunflower varieties (XKZ6 and SH363) were selected to investigated the abundance of *Lygus pratensis* with different varieties and planting dates, and cotton was investigated at the same time. According to previous habit, sunflower and cotton were all investigated 100 plants each time. But ignore the impact of plant size, this is indeed the negligence of our work.**

**And on the basis, we investigated the population of *Lygus pratensis* on sunflower(XKZ6) and cotton with the same size in 2017. The result showed that, compared to cotton, sunflowers have more attractive ability to *Lygus pratensis*. But this data has already appeared in another submitted paper.**

（*t*=14.83，*df*=1,3，*P*=0.0007）

Population density（ per m2）

|  | cotton | sunflower |
| --- | --- | --- |
| 2017-06-01 | 0.166667 | 1.83333 |
| 2017-06-05 | 0.583333 | 9.66667 |
| 2017-06-10 | 1.75 | 10.1667 |
| 2017-06-15 | 1.41667 | 11.5833 |
| 2017-06-20 | 1.08333 | 4.33333 |
| 2017-06-25 | 1.08333 | 4.16667 |
| 2017-06-30 | 0.25 | 3.25 |
| 2017-07-05 | 0.916667 | 3.91667 |
| 2017-07-10 | 1.16667 | 3.41667 |
| 2017-07-15 | 0.833333 | 1.25 |
| 2017-07-20 | 0.416667 | 0.833333 |
| 2017-07-25 | 0.25 | 0.0833333 |
| 2017-07-30 | 0.0833333 | 0 |
| 2017-08-04 | 0.25 | 0 |
| 2017-08-09 | 0.25 | 0 |
| 2017-08-14 | 0.166667 | 0 |

Population dynamics of *Lygus pratensis* adults on sunflower and cotton

4. Please check the data of Table 1 that in 2016, the p value of the adults, Nymphs were 0.0622 and 0.0430 respectively, but the p value of the total the adult and nymphs was 0.0167 not in range 0.0430~0.0622, is right?

**Reply: In response to your questions, we checked and analyzed the data again.**

**Compared with control treatments, population levels of *L. pratensis* adults and nymphs were markedly lower in cotton fields with sunflower borders. So the population levels of *L. pratensis* all individuals (nymphs and adults) were lower even more in cotton fields with sunflower borders, compared with control treatment. Therefore, the p value of the total adults and nymphs was 0.0167 not in range 0.0430~0.0622.**

We hope that the revised manuscript will meet with formal acceptance.

Again, we thank you and the reviewers for your time and effort in reviewing this manuscript.

Kindest regards.

Yours,

Renfu Zhang

Institute of Plant Protection

Xinjiang Academy of Agricultural Sciences

No.403, Nanchang Road

Urumqi, Xinjiang 830091, China
