# Supplementary material for: Field evaluation of sunflower as a potential trap crop of Lygus pratensis in cotton fields
Source: PLoS One. 2020 Aug 17;15(8):e0237318. doi: 10.1371/journal.pone.0237318 (PMC7430709; doi:10.1371/journal.pone.0237318)
Supplement: Attachment — Submitted filename: Response to Reviewers.docx [file pone.0237318.s003.docx]

Dear editors,

Thank you very much for your attention and careful consideration to our manuscript. We appreciate reviewers very much for their constructive comments and suggestions on our manuscript.

We have carefully considered your and referee’s suggestion and comments and revised our manuscript according to these precious comments. Those comments are invaluable for improving our paper. We have accordingly revised the manuscript carefully and hope that this new version could be accepted for publication in *PLOS ONE*. The main corrections and the responds to the comments point to point are given below:

**I. Responses to referee #2**

Thank you very much for your attention and careful consideration to our manuscript. Your constructive comments and suggestions are of great significance to improving the manuscript. Again, thank you for your time and effort in reviewing this manuscript.

**II. Responses to the comments and suggestions of Referee #3**

1. Line 35, integrated pest management (IPM), please capitalize the first letter of the first word.

**Reply: Thanks for the referee's suggestion. We have capitalized the first letter of the first word of integrated pest management (IPM) (Line 35).**

2. in text, there are some presentation like "F=70.88, df=6,14, P<0.0001", you can try like" F (6,14) =70.88, P=... "

**Reply: Thanks for the referee's suggestion. We have modified the format like your suggestion (Line 156-160 and 175-182).**

3.Line 160, "F=70.88，df=6,14, P<0.0001", the sampling times is 16 in range 6/3 to 8/17 in Figure 2, please check "df=14" is right?

**Reply: Thanks for the referee's suggestion.** **Population levels of *L. pratensis* adults among all seven treatments were analyzed using repeated measures ANOVA in 2015 and 2016, each of the above treatments was established in 3 field plots (i.e., replicates). it means that, 7 treatments, each treatment replicates 3 times, total of 21 plots in this test. Therefore, the correct is df=6,12. Through analysis again, found that this indeed a mistake.** **Thank you very much for your attention and careful review(Line 175-182).**

4. Also please check Ndf number of sampling period=8, you need to explain how to select data for analysis. please see times of sampling is 11 as showed in figure4.

**Reply: Thanks for the referee's suggestion. Difference in the abundance of *L. pratensis* (including adults, nymphs, and total population, as three counts) in cotton fields with/without sunflower strips (2016-2017) were analyzed by a repeated-measures analysis of variance (ANOVA) using SAS PROC MIXED, and the means were compared by the least significant difference test (LSD). In 2016 and 2017, *L. pratensis* abundances in both cotton fields were below the existing economic thresholds for this pest (i.e., 20 bugs per 100 cotton plants at blossoming stage; Wang et al., 2016) after middle July. Hence, only population abundance records from early June to mid-July were included in the repeated-measures ANOVA and non-paired t-tests, select sampling time 8 times for statistical analysis.** **Detailed explanation showed in the statistical analysis (line 134-147).**

We hope that the revised manuscript will meet with formal acceptance.

Again, we thank you and the reviewers for your time and effort in reviewing this manuscript.

Kindest regards.

Yours,

Renfu Zhang

Institute of Plant Protection

Xinjiang Academy of Agricultural Sciences

No.403, Nanchang Road

Urumqi, Xinjiang 830091, China
